# Injectable In Situ Gelling System for Sustained Nicotine Delivery as a Replacement Therapy for Smoking Cessation

**DOI:** 10.3390/gels8020114

**Published:** 2022-02-12

**Authors:** Eileen Hulambukie, Hani Abdeltawab, Sanjukta Duarah, Darren Svirskis, Manisha Sharma

**Affiliations:** School of Pharmacy, Faculty of Medical and Health Sciences, The University of Auckland, Auckland 1142, New Zealand; ehul368@aucklanduni.ac.nz (E.H.); h.abdeltawab@auckland.ac.nz (H.A.); s.duarah@auckland.ac.nz (S.D.); d.svirskis@auckland.ac.nz (D.S.)

**Keywords:** nicotine, sustained release, prolonged release, poloxamers, design expert, factorial design, gelation temperature, rheological properties

## Abstract

Nicotine replacement therapy (NRT) is widely used to limit the withdrawal symptoms associated with cigarette smoking cessation. However, the available NRT formulations are limited by their short release profiles, requiring frequent administrations along with local side effects. Thus, the objective of this study is to develop an NRT formulation that offers prolonged, sustained nicotine release. Thermoresponsive in situ gelling systems containing nicotine were prepared using poloxamer 407 (P407) and poloxamer 188 (P188). The system was optimized using a three-factor, two-level full factorial design (2^3^). A formulation composed of P407 (20% *w/w*), P188 (5% *w/w*), and loaded with nicotine (0.5% *w/w*) exhibited sol-to-gel transition at a suitable temperature close to physiological temperature (30 °C). The rheological analysis demonstrated a Newtonian-like flow at room temperature, suggesting ease of administration via injection, and semisolid gel status at physiological temperature. The optimized formulation successfully sustained nicotine in vitro release over 5 days following single administration. The findings suggest that poloxamer based in situ gelling systems are promising platforms to sustain the release of nicotine.

## 1. Introduction

Cigarette smoking is a worldwide health issue responsible for seven million deaths per year [1,2,3]. Cigarette smoke contains nicotine and a complex mixture of potential toxic substances such as carbon monoxide, polycyclic aromatic hydrocarbons, and oxidant gases [4]. Nicotine dependency is the major driving force towards excessive cigarette consumption, which is linked to multiple local adverse effects such as increased salivation, burning sensation in the mouth and throat, abdominal pain, nausea, and vomiting [5]. Additionally, cigarette smoking increases cardiotoxicity risk and can cause serious systemic side effects such as tremors, cyanosis, prostration, dyspnea, convulsion, and even coma [5].

There is an increasing global interest in decreasing the number of cigarette smokers, attributed to the health complications of smoking. Yet, smoking cessation is associated with multiple withdrawal symptoms such as craving to smoke, restlessness, lower concentration, and trouble sleeping. Nicotine replacement therapy (NRT) has been introduced to reduce withdrawal symptoms and assist in smoking cessation. They also help in avoiding the side effects caused by other chemicals present in the cigarette smoke [4,5]. Pharmacologically, NRT acts by stimulating the nicotine receptors in the brain, thereby releasing dopamine in the nucleus domain with a potential to reduce the withdrawal symptoms of nicotine and minimize the urge to smoke [6]. Several NRT formulations are available in the market, such as gum, sublingual tablets, lozenges, and nicotine patches. Yet, they are challenged by their short release profile, requiring frequent administration. Therefore, a sustained delivery option that could provide improved delivery for a longer period and minimize the local side effects would be a valuable therapeutic option for smoking cessation.

**Figure 1:**
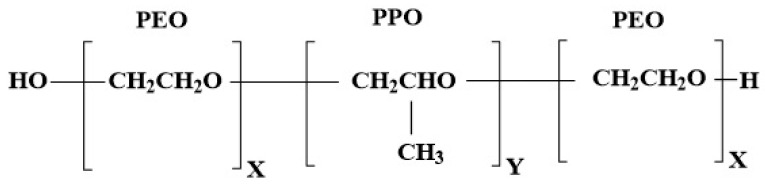
Representation of chemical structure of poloxamer; P407 x = 100, y = 68 and P188 x = 76, y = 29 (16).

Thermoresponsive in situ gelling systems, such as poloxamers, undergo sol-to-gel transition in response to temperature variations [7]. Poloxamers are Food and Drug Administration (FDA) approved biocompatible and biodegradable polymers. They allow easy administration via injection, as they are liquid at room temperature and form a gel depot at body temperature (37 °C), thus enabling the slow release of the incorporated drug. Poloxamers are non-ionic amphiphilic triblock copolymers of polyethylene oxide-polypropylene oxide-polyethylene oxide (PEO-PPO-PEO) (Figure 1), and can encapsulate both water-soluble and lipid-soluble drugs [8]. Modifying the ratio of hydrophobic (PPO) to hydrophilic (PEO) components plays a significant role in altering the formulation rheological, mechanical, and drug release properties [9]. Hence, a poloxamer based in situ gelling system is a potential delivery platform that can be utilized to overcome some of the problems encountered with the current delivery systems. Another advantage of using poloxamers over other delivery systems is the involvement of fewer manufacturing steps and cost savings.

**Figure 2:**
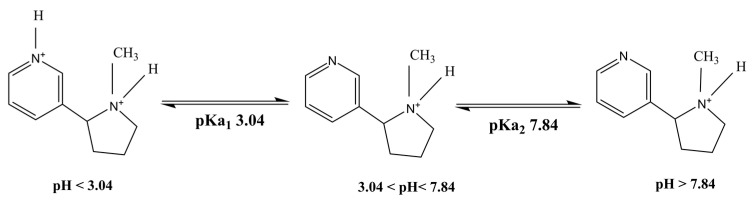
Representation of chemical structure of nicotine with different charged species [10].

Nicotine (Figure 2) is an alkaloid found in tobacco plants (*Nicotiana tabacum*). Nicotine is highly hydrophilic and lipophilic, possessing two potential ionizable functional groups in its chemical structure with pKa’s of 3.04 and 7.84, respectively [10]. Nicotine is widely used as replacement therapy for smoking cessation to minimize addiction and side effects. It easily crosses the mucosal membranes due to its freely available base form [10].

Hence, the main focus of this study is to explore the potential of poloxamer based in situ gelling systems for sustained delivery of nicotine for prolonged duration.

## 2. Results and Discussion

### 2.1. Screening of Poloxamer Concentrations

A three-factor, two-level (2^3^) full factorial experimental design (Design-Expert 11 software) was used to study the effect of the formulation components on the sol-to-gel transition (gelation) temperature. In the first screening, various concentrations of P407 alone and in combination with P188 were studied (Table 1). In line with the literature, formulation containing P407 below 15% did not show sol-to-gel transition, even when subjected to temperatures above 50 °C [11,12,13]. On the other hand, P407 alone at 20% concentration has shown sol-to-gel transition at room temperature (<25 °C), suggesting its unsuitability for the intended purpose [13]. Of note, a combination of P407 and P188 demonstrated sol-to-gel transition within the desired temperature range of 28–35 °C suitable for intramuscular applications.

In the second screening test, the concentration range of P407 was readjusted to 15–20% *w/w* (Table 2). An inverse relationship was observed between the P407 concentration and gelation temperature [12,13,14], while a direct relationship was observed between the P188 concentration and the sol-to-gel transition temperature. This may be due to the relatively hydrophobic nature of P407 and the relatively hydrophilic nature of P188 [8,12]. Of note, both blank and nicotine loaded formulations demonstrated comparable gelation temperatures, suggesting a minor effect of nicotine presence on the thermoresponsive properties of poloxamer gels. Therefore, nicotine concentration was omitted in the further optimization study.

Furthermore, it was revealed that the variables A (P407%) and B (P188%) had greater influence on the gelation temperature, with a standardized effect of 11.88 and 13.63, and percentage contribution of 35.75 and 47.07, respectively (Table 3). This implies that variable B had greater influence on increasing the gelation temperature than variable A, whereas nicotine concentration or interactions between variables had a limited effect on the gelation temperature. The main effect and percentage contributions of the formulation variables are shown in Table 3.

**Figure 3:**
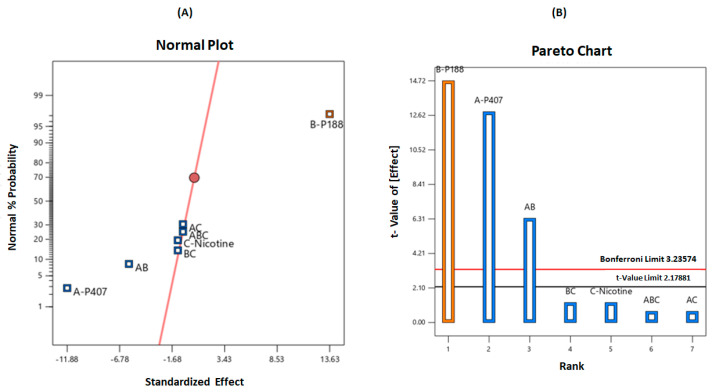
(**A**) Normal probability plot and (**B**) Pareto chart illustrating the significant variables on gelation temperature.

A normal plot of parameters was utilized to screen for the significant variables, as illustrated in Figure 3A. From the three tested independent variables, the factors and interactions deviating from the straight line were considered to have a significant effect on the gelation temperature. It is demonstrated on the plot that the concentrations of P407 (A), P188 (B), and the combinations of poloxamers (AB) were implicated as greatly influencing the response (gelation temperature). In addition, the Pareto chart also illustrates the statistical significance of each variable (Figure 3B). The Pareto chart represents the value by outlining the two limits, namely the Bonferroni limit line (t value of effect = 3.236) and the t limit line (t value of effect = 2.179). The variables and interactions of components with a t value above the Bonferroni line are identified as highly significant. Those between the Bonferroni line and the t limit line are thought to have significantly affected the response. In contrast, the variables and interactions with a t value of effect below the t limit line are considered as statistically insignificant and can be omitted for further analysis.

After determining the main effects on the response, ANOVA was then performed to identify the significant factors, with a *p*-value less than 0.05. The results showed that the model passed the significance test with a probability of less than 0.05 (>95% confidence). The two factors, namely P407 (A) and P188 (B), demonstrated a *p*-value less than 0.05 and significantly affected the sol-to-gel transition temperature. Moreover, the interaction between A and B was also observed to significantly affect the response. Thus, from this analysis, the non-significant variable, nicotine concentration (C), was not included in the optimization study. Furthermore, in this analysis, the model curvature appeared to be statistically significant with an F-value and *p*-value of 23.94 and 0.0005, respectively. With this outcome, a central composite design was employed for the further optimization of response.

#### Optimization and Evaluation

After the screening phase, a central composite design was employed to optimize and evaluate the results as suggested by the factorial design. The formulation variables selected were based on the results obtained from the factorial design. The poloxamer concentration range was selected based on the gelation temperature from the second screening. The software generated 13 runs of preparations, as shown in Table 4, with gelation temperature obtained from the experiments conducted. From the final analysis, the program suggested some formulations and predicted their responses containing a probability factor named “desirability” that ranged between 0 and 1. ANOVA was applied to estimate the significance of the model (*p* < 0.05), and the model F-value (<100) implies that the model is significant. Similarly, the fit statistics predicted the R² value to be in reasonable agreement with the adjusted R² value (i.e., the difference is less than 2). The adequate precision measuring the signal to noise ratio was also desirable, and thus, the model can be used to navigate the design space between formulation variables.

**Figure 4:**
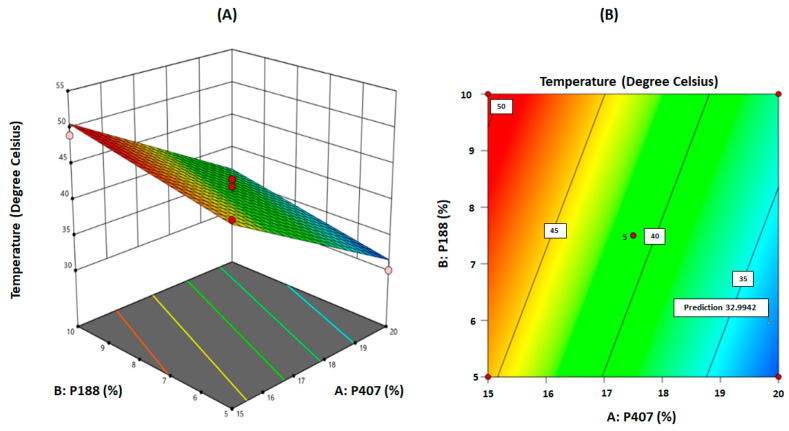
(**A**) Three-dimensional surface plot for gelation temperature as a function of the formulation variables. (**B**) Contour plot for gelation temperature as a function of the formulation variables (‘5’ refers to five replications on the centre point).

As shown in Table 5, the F-value of 39.75 implies that the model is significant. There is only a 0.01% chance that an F-value this large could occur due to noise. The *p*-values less than 0.05 indicated that the model terms are significant. In this case, A and B are significant model terms, as shown in Table 5. The predicted R² of 0.7748 is in reasonable agreement with the adjusted R² of 0.8659 (i.e., the difference is less than 0.2). Adequate precision measures the signal to noise ratio. A ratio greater than four is desirable. Thus, this model can be used to navigate the design space to accommodate variabilities. The model can also be represented graphically as a contour plot or a three-dimensional surface plot describing the response as a function of the two factors, A: P407 and B: P188 concentrations, as shown in Figure 4A,B, respectively. It can be seen from the graphs that the desired response can be achieved with a high concentration of P407 (20%) and a low concentration of P188 (5%). This could be the reason why a curvature is not observed in the surface plot, even though it was detected in the design models. It implies that if both variables were required in high concentration to achieve the desired response, a curvature could be observed in the surface plot [14]. Additionally, the two-dimensional contour plots relating to variables A and B (interaction between P407 and P188) were found to be linear, indicating the absence of interactions between these two variables [14]. From several solutions derived from numerical optimization, few formulations were selected with an ideal desirability.

### 2.2. Thermoresponsive Gel Preparation

The composition and gelation temperatures of the selected formulations prepared are shown in Table 6. As presented, the obtained gelation temperatures were in line with those predicted by the model. The inclusion of various nicotine concentrations in this study had a minor influence on the gelation temperature, probably due to the presence of two ionizable functional groups in its structure [10]. Ionizable aqueous soluble drugs tend to stay in the interconnected aqueous channels within the gel matrix, with minimal effect on micellar assembly and entanglement [9]. As shown, all formulations exhibited sol-to-gel transition at a temperature range of 28 to 35 °C, suggesting their suitability for the intended purpose. Formulations F1 to F3 showed gelation between 32 and 34 °C (Table 6), whereas formulation F4 demonstrated sol-to-gel transition at 30 °C. The developed formulation could ultimately find clinical application following administration via injection into a peripheral limb. As peripheral limbs can be considerably cooler than core temperature (37 °C), we selected 30 °C as the lowest sol-to-gel transition temperature that would be acceptable. Sol-to-gel transition temperatures higher than this would risk the formulation not gelling, or only gelling slowly, in the body with much faster release and a loss of any sustained release. Hence, we selected formulation F4, loaded with nicotine (0.5%) (F6), for further in vitro characterization involving mechanical and in vitro drug release studies.

### 2.3. Rheological Studies

**Figure 5:**
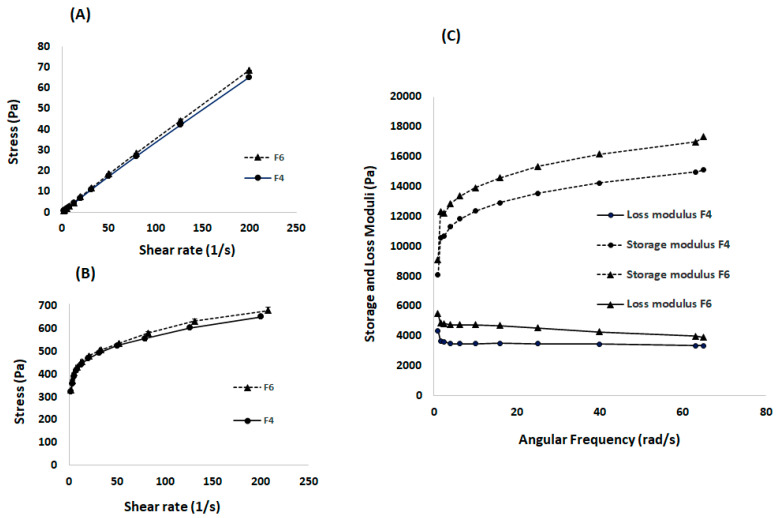
Rheological properties, showing (**A**) Flowability at 20 °C indicating a Newtonian flow behaviour, (**B**) Flowability at 37 °C indicating a pseudoplastic flow behaviour for both formulations: blank (F4) and nicotine loaded (F6), and (**C**)Viscoelasticity measurements at 37 °C demonstrating an elasticity with loss modulus having dominancy over the storage modulus for both formulations.

The rheological properties of the developed formulations help predict their ease of injectability (ability to be administered using a syringe and needle) at room temperature, and the in vivo performance after being injected into the body [15]. The viscosity of the formulation was measured as a function of shear rate against different shear stresses to determine the flowability at 20 °C (Figure 5A) and 37 °C (Figure 5B). A linear relationship between shear stress and shear rate was obtained at 20 °C, suggesting a Newtonian flow. On the other hand, a pseudoplastic flow was demonstrated at 37 °C, indicating the gel status at test conditions. This is desirable as it enables ease of syringeability (withdrawal from vial to syringe) and injectability at room temperature [16]. The solid-like status at 37 °C suggests the formation of a drug gel depot with a potential to sustain drug release. Of note, there was no significant difference in the flow behaviour between F4 and F6, indicating that the nicotine has a limited effect on the rheological properties, which could be attributed to its high aqueous solubility as explained above. Figure 5C shows the viscoelastic behaviour of the blank formulation (F4) and formulation loaded with nicotine (F6) over a range of angular frequencies at 37 °C. As presented, both formulations exhibited significantly higher storage modulus (G′) as compared to their loss modulus (G″) at all tested frequencies, suggesting their gel status at that temperature [17]. The greater elasticity of the gel could retard gel erosion, leading to a sustained release profile.

### 2.4. Measurement of Mechanical Properties

#### 2.4.1. Gel Strength

Gel strength and hardness of the formulations (F4 and F6) were examined to determine the gel mechanical properties at 37 °C. As presented in Table 7, both formulations demonstrated comparable mechanical properties, suggesting a limited effect of nicotine on gel microstructure.

#### 2.4.2. Injectability

The injectability of the formulations was investigated to ensure the suitability of the developed formulations for intramuscular administration. As presented in Table 7, a slight variation in injectability was observed between the blank (F4) and nicotine loaded (F6) formulations. It was observed that once the formulation started to flow through the needle, the force remained almost steady for the blank formulation until the plateau force was reached, followed by a reduction in the end constrain force. The nicotine loaded formulation indicated a rather lower expelling force than the blank formulation, consistent with its lower gel strength and hardness properties. This observation is similar to previous studies where a stronger gel required a greater injectability force than a weaker gel [18].

Formulations consisting of in situ gelling systems are often highly viscous and require large needles, such as an 18-gauge, to be easily administered, causing pain and discomfort to the patient [15,19,20]. As demonstrated, the optimized formulation was easily injected through a 21-gauge needle, suggesting less pain during administration.

### 2.5. Calibration Curve of Nicotine

**Figure 6:**
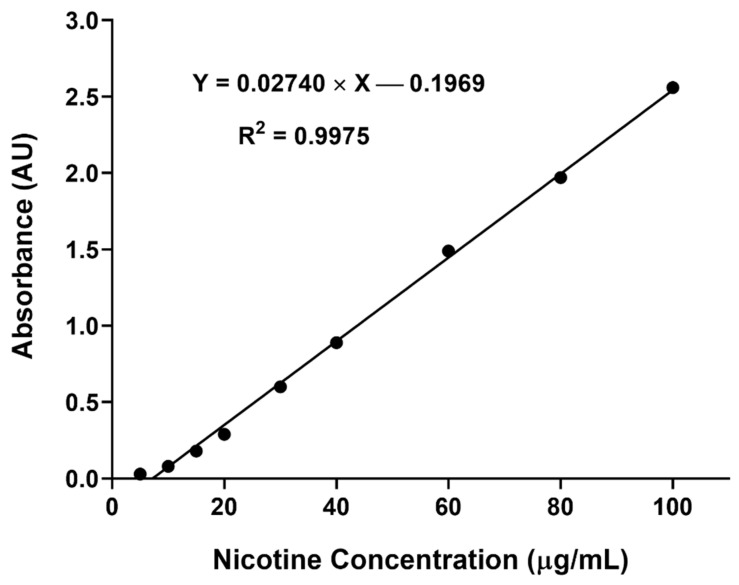
Nicotine calibration curve demonstrating acceptable linearity of absorbance at 258 nm against concentration over the range of 10 to 100 µg/mL (n = 3).

As shown in Figure 6, the response between the nicotine concentration and absorbance was linear in the concentration range of 10 to 100 µg/mL. The standard deviation of the slope and intercept obtained were low, with the determination coefficient (R^2^) exceeding 0.99, suggesting the validity of the developed method for intended purpose [21].

### 2.6. In Vitro Release Studies

**Figure 7:**
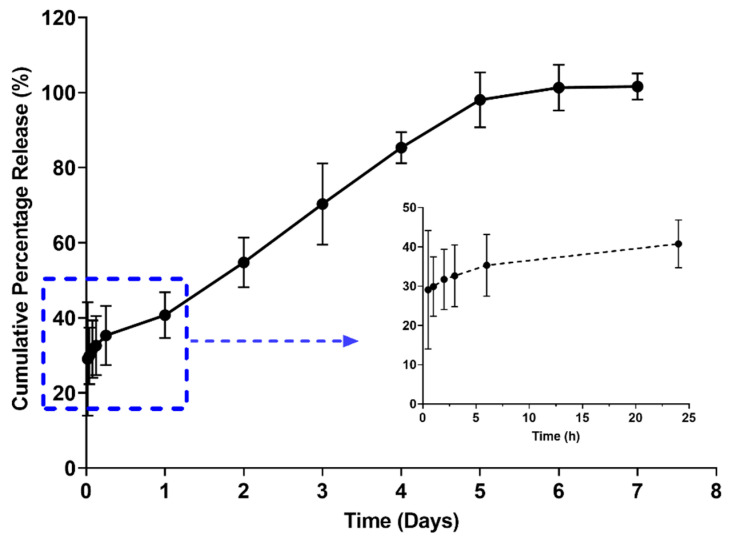
In vitro drug release profile demonstrating a sustain release over a period of 7 days with initial burst release as shown in insert (n = 3).

As shown in Figure 7, the optimized formulation demonstrated a sustained release of nicotine over a week with an initial burst release of ~30% within the first hour. This could be explained by a lag time between injection and gel formation [22]. This was followed by a slower and sustained release of nicotine attributed to the formation of a highly entangled matrix, hindering the diffusion of nicotine to the external environment [7,23,24,25]. Of note, the drug release rate was linear over a period of 5 days and reached a plateau beyond 5 days. The findings suggest the potential of poloxamers for sustaining nicotine release. Yet, further studies are required to fully understand the release mechanism and to study the in vivo performance of the developed formulation.

In comparison to the available NRT formulation, a sustained release intramuscular formulation is of advantage as it will improve patient compliance by reducing frequent administration, as well as improving bioavailability. Moreover, the initial burst release observed in this study could be beneficial, as it will provide a rapid systemic absorption of nicotine comparable to cigarette smoking [26], thus providing patient satisfaction and alleviating the urge for frequent nicotine intake from cigarettes to supply the immediate feeling of satisfaction. In addition, the formulation could give sustained release over a longer period to maintain nicotine systemic concentration, and minimize nicotine dependency and side effects from smoking cigarettes.

## 3. Future Perspectives

The present study investigated the potential of poloxamers for sustaining the release of nicotine in NRT. Future studies might consider the physical blending of the optimized formulation with additives, such as sodium chloride, sodium alginate, and methylcellulose, to modulate the initial burst release of nicotine [9]. A high-performance liquid chromatography might be used in analyzing the release samples, as it offers higher sensitivity and specificity compared to UV spectrophotometry [27]. Finally, the in vitro data from this study could be the basis for further in vivo investigation of a sustained release formulation for nicotine delivery.

## 4. Conclusions

This study is the first to employ poloxamer based in situ gelling systems for sustaining nicotine delivery as an NRT option. A quality by design approach was successfully used in developing an optimized poloxamer based in situ gelling system, with the ability to provide sustained release of nicotine over a period of 5 days. The study demonstrated that the gelation temperature can be adjusted by modifying the matrix composition. Mechanical studies showed the suitability of the optimized formulation for parenteral administration. This is the first poloxamer-based formulation to offer a sustained nicotine release, with potential clinical benefits in NRT. 

## 5. Materials and Methods

### 5.1. Materials 

Nicotine (≥99%) used in this study was obtained from Thermo Fisher Scientific New Zealand. Poloxamer 407 (P407) (Molecular weight ~12,600 g/mol), Kolliphor (P188)

(Molecular weight ~7680–9510 g/mol) and phosphate-buffered saline (PBS) tablets were purchased from Sigma-Aldrich (St. Louis, MO, USA). Water was obtained from a CFOF 01205 Milli-Q water purification system (Millipore, Burlington MA, USA). All other reagents and solvents used were of analytical grade.

### 5.2. Methods

#### 5.2.1. Experimental Design for Screening and Optimization Study

The design of experiments (DoE) in this study was constructed using Design-Expert^®^ software (version 11, Stat-Ease, Inc., Minneapolis, MN, USA). A two-level, three-factorial (2^3^) design approach was adopted to screen the poloxamer and drug concentration for the preparations of in situ gels. In the screening phase, the independent parameters screened were concentration of P407, concentration of P188, and concentration of nicotine drug. The level for each formulation variable was set based on the studies done in the past [12,22]. The selected response for this study design was sol-to-gel transition temperature (gelation temperature). The variables were screened at two levels: high and low, which are represented by transform codes −1 and +1, respectively (Table 8). The 2^3^ factorial design statistical software generated various compositions of P407, P188, and nicotine drug concentrations comprising of 20 experimental runs of in situ gel formulations. The number of runs included duplicates at centre points to minimize experimental errors and check for the response curvature.

The experiments were carried out in triplicate in a random order as suggested by the software. As shown in Table 8, each variable employed in the design was given a high-level and low-level value with the generation of centre point values by the software. Based on the results (responses) obtained from the first screening, P407 concentration was readjusted for the second screening (Table 8).

#### 5.2.2. Optimization of Poloxamer Concentrations

A central composite response surface design was employed for the optimization study. The critical variables that greatly influenced gelation temperature, as identified in the screening phase, were further optimized using the central composite design. In this study, the variables P407 and P188 were selected as independent variables and examined at five different concentrations. The main effects of the variables on the response were derived from the Pareto chart. The percentage contribution towards the response was obtained from the standardized effect and by minimizing the sum of square values. A total number of 13 runs of experiments was done with five replicates at the centre points. Statistical parameters, such as Fisher F-value using F-test, determination coefficient (R^2^), adjusted R^2^ (R^2^ adj), and the R^2^ of prediction (R^2^ pred), were utilised to select the best regression model among the linear, two-factor interaction model and quadratic model, and the data analysis was performed using ANOVA by testing for significance. Contour plots were used to display the relationship and interactions between the variables and the responses, and the optimal levels were derived from the plots.

#### 5.2.3. Thermoresponsive In Situ Gel Preparation

Thermoresponsive in situ gels were prepared by the cold method as previously described [16]. Briefly, a predetermined weight of P407, P188, and nicotine, as suggested by the DoE design, were added in PBS (pH 7.4) and stirred at 150 rpm overnight under 4 °C until a clear homogenous solution was obtained.

#### 5.2.4. Sol-to-Gel Transition (Gelation) Temperature

The gelation temperature of the prepared formulations was determined using a Discovery HR-2 rheometer (TA instruments, Melbourne, Australia), equipped with a 40 mm stainless steel parallel plate and temperature-controlled Peltier plate. The temperature ramp (20–50 °C) was performed at a heating rate of 2 °C/min to measure the sample viscosity as a function of temperature. The sample was considered gelled at the mid-point of the increasing viscosity between the liquid and gel state [28]. The experiments were conducted in triplicate.

#### 5.2.5. Rheological Studies

The rheology experiments (involving viscoelasticity and flow behaviour) were performed using the HR-2 rheometer. The frequency sweeps were carried out to observe the viscoelastic properties of the gel. An angular frequency range of 1–65 rad/s was selected, and the samples were studied at 37 °C. All the experiments were performed in the linear viscoelastic region of the gels (applied stress 0.02 Pa) [22]. The flowability measurements were determined as a function of shear stress (Pa) against shear rate (1/s) in the range of 2–200 s^−1^ at both 20 °C and 37 °C [23].

#### 5.2.6. Measurement of Gel Mechanical Properties

- Gel strength

The gel strength (compressibility) and hardness (firmness) were determined using a texture analyser (Stable Microsystems, UK) with a cylinder probe (10 mm). The formulation (15 mL) was transferred into vials and maintained at 37 °C for 20 min to ensure gel formation. The developed gel was then compressed to a depth of 10 mm with a trigger force of 2 gf at a rate of 2 mm/s, and the force required for penetrating the gel was measured. Each formulation was tested in triplicate. Hardness and gel strength were derived from the resultant force-time plot using the Exponent 32 software [22].

- Injectability

The prepared formulations were tested for injectability using a universal syringe rig (A/USR) attachment (Stable Microsystems, Surrey, UK), a 10-mL syringe, and a 21-gauge (G) needle at a speed of 5 mm/s with a contact force of 50 gf, return speed of 20 mm/s, and return distance of 50 mm. The parameters measured were (i) stiction force, the force required to overcome the resistance force of the syringe’s plunger, (ii) plateau force, the force required to maintain plunger movement to expel the content from the syringe, and (iii) the end constraint, which is the syringe plunger compression against the end of the syringe body [18]. The study was performed in triplicate.

#### 5.2.7. Preparation of Calibration Curve for Nicotine

Nicotine was quantified using a UV-Vis spectrophotometric method. Initially, the nicotine stock solution (1 mg/mL) was prepared in PBS (pH 7.4). The serial dilution (5, 10, 20, 40, 80 and 100 µg/mL) of the stock solution was prepared in the same diluent, and the absorbance of the prepared solutions was measured at 258 nm. The absorbance was plotted against the concentration, and the linear regression method was applied to determine the linearity and the determination coefficient (R^2^). For the absorbance greater than 1, the samples were diluted and measured again, and the dilution factor was considered when plotting the calibration curve. The experiment was performed in triplicate to get the standard deviation for the nicotine concentrations.

#### 5.2.8. In Vitro Drug Release Study

In vitro drug release was carried out using falcon tubes in PBS medium (pH 7.4) at 37 °C. Specified amounts (2 mL) of formulation were injected into the prewarmed (37 °C) PBS (10 mL) contained in the falcon tube. Tubes of gelled formulation were then placed in the shaking water bath at 35 rpm and maintained at 37 °C. At predetermined time points, 1 mL of samples was withdrawn for analysis and the volume was replaced with pre-warmed PBS solution.

## Figures and Tables

**Table 1:** First screening design showing variables (in coded values) and responses.

		Variable 1	Variable 2	Variable 3	Response 1
Std	Run	A: P407 (%)	B: P188 (%)	C: Nicotine (%)	Experimentally Determined Gelation Temperature (°C)
9	1	−1	−1	+1	>50
7	2	+1	+1	−1	33
20	3	0	0	0	42
2	4	−1	−1	−1	>50
13	5	−1	+1	+1	>50
8	6	+1	+1	−1	30
12	7	+1	−1	+1	24
17	8	0	0	0	43
10	9	−1	−1	+1	>50
16	10	+1	+1	+1	30
14	11	−1	+1	+1	>50
11	12	+1	−1	+1	21
4	13	+1	−1	−1	21
19	14	0	0	0	43
6	15	−1	+1	−1	>50
18	16	0	0	0	>50
15	17	+1	+1	+1	31
3	18	+1	−1	−1	24
1	19	−1	−1	−1	>50
5	20	−1	+1	−1	>50

(A) P407 concentration (%); Low (−1) 10%, Centre (0) 15%, High (+1) 20%, (B) P188 concentration; Low (−1) 0%, Centre (0) 5%, High (+1) 15% (%), (C) Nicotine concentration (%); Low (−1) 0%, Centre (0) 0.25%, High (+1) 0.5%.

**Table 2:** Second screening design showing variables (in coded values) and responses.

Std	Run	Variable 1 A: P407 (%)	Variable 2 B: P188 (%)	Variable 3 C: Nicotine (%)	Response 1 Experimentally Determined Gelation Temperature (°C)
11	1	+1	−1	+1	20
17	2	0	0	0	37.5
14	3	−1	+1	+1	48
15	4	+1	+1	+1	26
7	5	+1	+1	−1	29
13	6	−1	+1	+1	42
5	7	−1	+1	−1	46
9	8	−1	−1	+1	26
6	9	−1	+1	−1	46
18	10	0	0	0	34
10	11	−1	−1	+1	26
20	12	0	0	0	32
1	13	−1	−1	−1	25
2	14	−1	−1	−1	27
8	15	+1	+1	−1	30
16	16	+1	+1	+1	26
3	17	+1	−1	−1	20
19	18	0	0	0	36
12	19	+1	−1	+1	20
4	20	+1	−1	−1	20

(A) P407 concentration (%); Low (−1) 15%, Centre (0) 17.5%, High (+1) 20%,, (B) P188 concentration (%); Low (−1) 0%, Centre (0) 5%, High (+1) 15%,, (C) Nicotine concentration (%); Low (−1) 0%, Centre (0) 0.25%, High (+1) 0.5%.

**Table 3:** Effects of each single variable and its interactions in variable screening.

	Response (Gelation Temperature)	
Variable	Standardized Effect	Contribution (%)
A	11.88	35.75
B	13.63	47.07
C	−1.13	0.32
AB	−5.88	8.75
AC	−0.63	0.10
BC	−1.13	0.32
ABC	−0.63	0.10
Curvature	4.53	5.20

(A) P407 concentration (%), (B) P188 concentration (%), (C) Nicotine concentration (%).

**Table 4:** Optimization design showing variables (in coded values) and responses.

Std	Run	Variable 1 A: P407 (%)	Variable 2 B: P188 (%)	Response 1 Experimentally Determined Gelation Temperature (°C)
7	1	0	−2.04	37
5	2	−2.04	0	49
2	3	+1	−1	30
4	4	+1	+1	32
10	5	0	0	42
12	6	0	0	42
11	7	0	0	43
9	8	0	0	43
8	9	0	+2.04	48
13	10	0	0	40
3	11	−1	+1	49
6	12	+2.04	0	33
1	13	−1	−1	46

**Table 5:** ANOVA for the response optimization.

Source	Sum of Squares	Df **	Mean Square	F-Value	*p*-Value
Model	439.62	2	219.81	39.75	˂0.0001 *
A	386.8	1	386.8	69.94	˂0.0001 *
B	52.82	1	52.82	9.55	0.0114 *
Lack of fit	49.05	5	9.81	6.54	0.0464 *
Pure Error	6	4	1.5		
Cor Total	494.92	12			

(A) P407 concentration (%), (B) P188 concentration (%). * statistically significant *p* ˂ 0.05, R² = 0.8883, Adjusted R² = 0.8659, Predicted R² = 0.7748, ** degree of freedom.

**Table 6:** Gelation temperatures for the optimized formulation.

Formulations	Factor 1 A: P407 (%*w/w*)	Factor 2 B: P188 (%*w/w*)	Factor 2 C: Nicotine (%*w/w*)	Response 1 Experimentally Determined Gelation Temperature (°C)
F1	19.83	5.95	0	34
F2	19.83	5.95	0.25	32
F3	19.83	5.95	0.5	32
F4	20	5	0	30
F5	20	5	0.25	30
F6	20	5	0.5	30

**Table 7:** Mechanical properties of the optimized formulations.

Formulation	Hardness (gf)	Gel Strength (gf.s)	Injectability		
			Stiction Force (gf)	Plateau Force (gf)	End Constraint (gf)
	(Mean ± SD)				
Blank (F4)	28.5 ± 3.4	113.6 ± 0.9	557.7 ± 50.6	557.9 ± 50.4	534.5 ± 133.5
Nicotine loaded (F6)	27.9 ± 3.2	112.6 ± 1.8	535.2 ± 143.8	521.7 ± 220.8	517.5 ± 98.1

**Table 8:** Factorial design variables and experimental conditions.

Factors	1st Screening			2nd Screening		
	Level Used, Actual (Coded)			Level Used, Actual (Coded)		
	Low (−1)	Centre (0)	High (+1)	Low (−1)	Centre (0)	High (+1)
P407 (%)	10%	15%	20%	15%	17.5%	20%
P188 (%)	0%	5%	15%	0%	5%	15%
Nicotine (%)	0%	0.25%	0.5%	0%	0.25%	0.5%

## Data Availability

Not applicable.
